# Influence of Cardiorespiratory Fitness on *PPARG* mRNA Expression Using Monozygotic Twin Case Control

**DOI:** 10.1155/2015/538732

**Published:** 2015-03-24

**Authors:** Marcos Roberto Queiroga, Ricardo Augusto Barbieri, Sandra Aires Ferreira, André Ducati Luchessi, Rosario Dominguez Crespo Hirata, Mario Hiroyuki Hirata, Eduardo Kokubun

**Affiliations:** ^1^Departamento de Educação Física da Universidade Estadual do Centro-Oeste (UNICENTRO), Rua Simeão Camargo Varela de Sá, 03, 85040-080 Guarapuava, PR, Brazil; ^2^Departamento de Educação Física da Universidade Estadual Paulista (UNESP), Avenida 24 A 1515, 13506-900 Rio Claro, SP, Brazil; ^3^Departamento de Análises Clínicas e Toxicológicas da Universidade Federal do Rio Grande do Norte (UFRN), Rua General Gustavo Cordeiro de Farias, 384, 59012-570 Natal, RN, Brazil; ^4^Departamento de Análises Clínicas e Toxicológicas da Universidade de São Paulo (USP), Avenida Professor Lineu Prestes, 580, 05508-000 São Paulo, SP, Brazil

## Abstract

The influence of cardiorespiratory fitness (VO_2_max) on anthropometric variables and* PPARG* mRNA expression was investigated. Monozygotic twin pairs aged 11–18 years were grouped into discordant (D) and concordant (C) high and low VO_2_max groups. VO_2_max was determined by progressive maximal exercise test on treadmill with gas exchange analysis. Body mass (BM), BMI, waist circumference (WC), triceps (TR), and subscapular (SB) skinfold thicknesses were measured. Twins from the discordant group had differences in VO_2_max values (D-high = 45.9 ± 10.0 versus D-low = 32.4 ± 10.6 mL·kg^−1^·min^−1^, *P* = 0.025), while no differences were found in the concordant group (C-high = 42.4 ± 9.2 versus C-low = 38.8 ± 9.8 mL·kg^−1^·min^−1^, *P* = 0.952). In discordant group, VO_2_max was negatively correlated with TR + SB (*r* = −0.540, *P* = 0.021) and positively correlated with* PPARG* expression in leukocytes (*r* = 0.952, *P* = 0.001). Moreover,* PPARG* expression was directly correlated with BM (*r* = 0.714, *P* = 0.047) and height (*r* = 0.762, *P* = 0.028). In concordant twins, VO_2_max was inversely correlated with BM (*r* = −0.290, *P* = 0.027), BMI (*r* = −0.472, *P* = 0.001), WC (*r* = −0.426, *P* = 0.001), and TR + SB (*r* = −0.739, *P* = 0.001). Twins D-high had 1.78-fold greater* PPARG* expression when compared with twins D-low (*P* = 0.048). In conclusion, the cardiorespiratory fitness may modulate* PPARG* expression in childhood and adolescence, independently of the genetic background.

## 1. Introduction

Peroxisome proliferator-activated receptors (*PPARs*) are involved in the regulatory response processes of lipid and glucose metabolisms, adipocyte differentiation, and inflammatory response [1]. The three peroxisome proliferator-activated receptors (*PPARs*) *α*, *β* (*δ*), and *γ* constitute a distinct subfamily in the superfamily of nuclear receptors. PPARs are ligand-regulated transcription factors that control gene expression by binding to specific response elements within promoters. Studies have shown that* PPARs* are involved in the physiopathology of complex metabolic disorders, such as those related to metabolic syndrome, resulting in atherosclerosis and cardiovascular diseases [2].

*PPARG* regulates the expression of numerous genes involved in controlling cellular energy homeostasis, lipid metabolism, insulin action, and adipocyte differentiation [3]. In a study using cell culture, Uruno et al. [4] showed the inhibitory effect of* PPARG* agonists, controlling blood pressure, on angiotensin-II induced aldosterone synthase expression and aldosterone secretion. An association of* PPARG* gene variants with insulin resistance, type 2 diabetes, obesity, and hypertension has been shown [5, 6]. Additionally,* PPARG* activation is associated with increased consumption of free fatty acids by skeletal muscle and adipose tissue, inhibition of the expression of proinflammatory genes (TNF*α*) that trigger atherosclerosis, and increased insulin sensitivity [7]. Atherosclerosis is usually preceded by endothelial dysfunction, whereas agonists have been reported to improve the function of these cells in type 2 diabetic patients [8] and nondiabetic patients with coronary artery disease [9].

Besides the role played by* PPARG* activation in regulating the metabolism of glucose and lipids, a possible regulatory role in the expression of proinflammatory adipokines has also been suggested [1, 6]. Additionally, the renal protective effect of* PPARG* agonists against nondiabetic renal disease [10] and anticancer activities of* PPARG* agonist was recently reported [11].

It has been also shown that* PPARG* variant (*PPARG* C1A) is related to effects on the effectiveness of aerobic exercise training to increase aerobic fitness and insulin sensitivity [12]. Moreover, results suggest that the mitochondrial function associated with aerobic fitness and insulin resistance is deeply affected by the expression of coactivators (PGC-1*α* and PGC-1*β*) of the* PPARG* [13]. Therefore,* PPARG* may be a promising candidate for regulation by maximal oxygen uptake [14].

The cardiorespiratory fitness as defined by maximal oxygen uptake (VO_2_max) is modulated largely by physical activity level and genotype. A low VO_2_max has been shown to be associated with glucose intolerance, insulin resistance, type-2 diabetes mellitus [15, 16], and downregulation of the* PPARG* mRNA expression from skeletal muscle and peripheral mononuclear cells [17, 18]. However, it has not been demonstrated whether association between VO_2_max and* PPARG* mRNA expression is influenced by familial or genetic background.

Nevertheless, studies involving the analysis of the interaction between physical activity and genetic aspects have increased considerably in recent years, but knowledge about the impact or effect of physical exercise on genome or highlighting the limitations of genetic background in physical performance is preliminary. It has been argued that the human genome has evolved over a period when high levels of physical activity were essential for survival, which supports the view that there is a link between exercise and gene expression regulation [19].

The direct influence of VO_2_max on* PPARG* expression has not been previously examined, regardless of genetic background, but it can be evaluated using the discordant monozygotic (MZ) twin model (cotwin case control study design). This experimental model allows estimating the effect of one discordant environmental factor [20, 21]. MZ twins have the same inherited genes, and if they differ in a particular trait, the difference can be considered to be due to environmental factors [22]. Using this model, the influence of VO_2_max can be estimated, isolated from additional covariates that usually confound other studies. The aim of this study was to investigate the influence of VO_2_max on* PPARG* mRNA expression using MZ twin model case control.

## 2. Materials and Methods

### 2.1. Subjects

Ninety-eight twin pairs who attended private and public schools of Rio Claro, São Paulo, were invited to participate in this study. Among the twin pairs, 53 were girls and 45 were boys aged from 11 to 18 years. In 31 twin pairs, one or both twins declined participation and 13 pairs were not found. Fifty-four twin pairs (35 girls and 19 boys) attended clinical and evaluation sessions. Parental testing by microsatellite analysis was performed as previously described [23] and revealed that the sample evaluated had 38 MZ twin pairs and 16 dizygotic (DZ) twin pairs. In the study, only MZ twin pairs were included (24 girls and 14 boys, 14.3 ± 2.1 years old; 51.4 ± 12.9 kg; 158.2 ± 10.6 cm). The cotwin control design is a powerful research methodology for studying the effects of environmental risk factors on the development of disease. In the case-control study, disease discordant pairs are examined for differences in antecedent exposures [24].

MZ twin pairs, their parents, and/or guardians were previously informed about the experimental procedures and provided written consent for participation. The intervention protocols were approved by the Research Ethics Committee (protocol no. 5093) of the São Paulo State University (UNESP), according to the norms of Resolution 196/96 of the National Health Council on research involving humans.

### 2.2. Anthropometric and Physical Fitness Measurements

Body mass and height were measured barefoot in light clothing to the nearest 100 g and 0.1 cm, respectively. Body mass index (BMI) was calculated by dividing body mass (kg) by height (m^2^). Waist circumference was measured in triplicate, midway between the lowest rib and the superior border of the iliac crest, with a flexible tape. The average of the 3 waist circumference measurements was used in all analyses. Skinfold thicknesses were measured at the triceps (TR) and subscapular (SB) on the right side of the body with a Harpenden skinfold calipers (Holtain Ltd., Bryberian, UK). Three sets of measurements were taken to the nearest 0.2 mm at each site and the mean of the three values was used. Anthropometric measurements were performed according to conventional procedures [25].

Work-conducted maximal exercise test with gas exchange analysis (spiroergometry) was performed using treadmill model ATL Super (Inbrasport, Porto Alegre, RS, Brazil) with 1% inclination, in the morning and afternoon periods from 9:00 a.m. to 11:30 a.m. and from 2:00 p.m. to 6:00 p.m. in a room temperature chamber maintained between 20 and 25°C. After a 5-minute adaptation period with treadmill at different speeds (4 to 7 km/h), the individuals remained five minutes on the treadmill at rest in standing position. At rest and during exercise test, respiratory minute volume (VE), oxygen intake (VO_2_), and carbon dioxide production (VCO_2_) were continuously recorded by assessing pulmonary gas exchanges (metabolic analyzer VO2000 MedGraphics Medical Graphics Corp., St. Paul, MN). The equipment was calibrated prior to the beginning of each individual test. Then, the test was performed using protocol that provided initial speed of 4 km/h with progressive increase in workload of 1 km/h per minute until exhaustion (19-20/20 on the Borg scale for perceived exertion or respiratory quotient (RQ = VCO_2_/VO_2_) of at least 1.1). Verbal encouragement was given in the attempt to obtain maximum physical exertion.

VO_2_max was collected breath by breath and the value adopted for analysis of data was recorded as the average oxygen uptake during 30 s that preceded test interruption. Heart rate was obtained by the Polar S810 heart rate monitor (Polar Electro, Finland). To apply the model of studies with MZ twin pairs (case-control model), the absolute intrapair difference in VO_2_max ≥ 10 mL·kg^−1^·min^−1^ was used to define twin pairs discordant for VO_2_max.

### 2.3. Blood Samples

Blood samples were collected from an antecubital vein using vacutainer tubes (Vacutainer Becton Dickinson Company, Plymouth, UK) exactly one week after cardiorespiratory fitness assessment (VO_2_max), from the twin pairs under their parents' care and authorization, at 7.30 a.m. after overnight fasting (10 to 12 h) and 30 min resting.

Approximately 150 *μ*L of blood was transferred to QIAcard FTA Spots (Qiagen, Valencia, USA) for DNA extraction and parental testing by microsatellite analysis. For mRNA expression, 4 mL of blood was transferred to Paxgene tubes (Qiagen, Valencia, USA) to further total RNA extraction.

### 2.4. Total RNA Isolation

Total RNA was isolated immediately after blood collection according to manufacturer's instructions with minor modifications using PAXgene Blood RNA kit (Qiagen, Valencia, USA). The entire procedure was carried out at room temperature. RNA purity was determined by spectrophotometry (A260/A280 ratio) using the equipment NanoDrop (NanoDrop Technologies, Inc., Wilmington, USA). RNA integrity was determined on BioAnalyzer (Agilent Technologies Inc., Santa Clara, USA) and RNA was regarded as intact when showing two distinct bands for 28S and 18S RNA.

### 2.5. Gene Expression Analysis by Real-Time PCR

The* PPARG* mRNA expression was measured by quantitative real-time PCR (q-PCR). cDNA was synthesized using the High Capacity Reagent (Applied Biosystems, Foster City, USA), according to manufacturer's protocol using the thermocycler Veriti 96 Well Thermal Cycler (Applied Biosystems, Foster City, USA), and it was stored at −20°C.

The cDNA from total RNA of peripheral blood leukocytes was amplified by qPCR using TaqMan amplifying system and ABI 7500 equipment (Applied Biosystems, Foster City, USA). Primers and fluorophore-labeled probes specific for* PPARG* and reference gene glyceraldehyde-3 phosphate dehydrogenase (GAPD) (Table 1) were designed using Primer Express (Applied Biosystems, Foster City, USA) and Primer Premier 5.0 (Premier Biosoft, Palo Alto, USA) software.

qPCR was performed under the following conditions: 20 *μ*L of final reaction volume containing TaqMan master mix 1 X (AmpliTaq DNA Polymerase, dNTPs with dUTP, AmpErase UNG) (Applied Biosystems, Foster City, USA), 300 nM of primers, and 200 nM of labeled probes. The PCR amplification program consisted of (1) one cycle of 2 min at 50°C (UNG activation), (2) one cycle of 5 minutes at 95°C (UNG inactivation); (3) 40 cycles of 15 sec at 95°C (denaturation); (4) 1 min at 60°C (hybridization and extension). The fluorescence signals emitted by fluorophores of TaqMan probes were detected by ABI Prism 7500 (Applied Biosystems, Foster City, USA). Data were analyzed using the System 7500 SDS software (Applied Biosystems, Foster City, USA) that generates half-log curves of amplification signals and Ct values. The Ct values were used to calculate the relative mRNA expression of* PPARG* compared to* GAPD* (reference gene) using the ΔCt. It is a calculated formula: ΔCt = (Ct_target  gene_ − Ct_reference  gene_).

### 2.6. Parental Testing by Microsatellite Analysis

Total DNA was extracted from whole blood samples in QIAcard FTA Spots with QIAamp 96 DNA Blood Kit (QIAGEN, Valencia, USA) according to manufacturer's recommendations. Microsatellite analysis was carried out by PCR using the Identifiler commercial kit, according to the manufacturer's instructions (Applied Biosystems, Foster City, EUA). This assay allows the genotyping of 15 multiple informative genetic markers (CSF1PO, D2S1338, D3S1358, D7S820, D8S1179, D13S317, D16S539, D18S51, D19S433, D21S11, D5S51, FGA, TH01, TPOX, VWF, and AMELXloci), which allows for the identification of monozygotic and dizygotic twins [23].

### 2.7. Statistical Analyses

Data were analyzed with the use of descriptive statistics as means and standard deviation. Normality of variables was assessed by Shapiro-Wilks test. For normally distributed variables, one-way analysis of variance for multiple comparisons followed by a Bonferroni's post hoc test was used to compare the means (anthropometric traits) between twin pairs with high and low VO_2_max and Wilcoxon's signed rank test for intrapair comparison of nonnormally distributed data (gene expression). Spearman's rank coefficient was used to investigate correlations between VO_2_max-discordant and concordant MZ twins. All statistical analyses were conducted using SPSS software version 20 (IBM SPSS), and alpha was set at *P* < 0.05.

## 3. Results

Twin pairs were distributed into discordant (10 girls and 8 boys aged 13.9 ± 2.2 years old) and concordant (38 girls and 20 boys aged 14.2 ± 2.1 years old) for VO_2_max, and within each group, cotwins were divided into high and low VO_2_max. Comparison of anthropometric characteristics for VO_2_max-discordant and concordant pairs is shown in Table 2. There were no differences (between and within group) in age and anthropometry characteristics (body mass, height, BMI, waist circumference, and sum of skinfold TR + SB). As expected, cotwins from the discordant group (D-high versus D-low) showed difference in VO_2_max values (32.4 ± 10.6 versus 45.9 ± 10.0 mL·kg^−1^·min^−1^, *P* = 0.025), while cotwins from the concordant group (C-high versus C-low) did not show difference in VO_2_max (38.8 ± 9.8 versus 42.4 ± 9.2 mL·kg^−1^·min^−1^, *P* = 0.952). The analysis also revealed that discordant low cotwins (D-low) showed marginal difference in VO_2_max values compared for concordant high twins (C-high) (32.4 ± 10.6 versus 42.4 ± 9.2 mL·kg^−1^·min^−1^, *P* = 0.050) (Table 2).

Correlation analysis for VO_2_max and* PPARG* mRNA expression with anthropometric characteristics in MZ twin's pairs discordant and concordant for VO_2_max is shown in Table 3. The VO_2_max was negatively correlated with the sum of skinfolds (−0.540, *P* = 0.021) and it had a strong positive correlation with* PPARG* mRNA expression (*r* = 0.952, *P* = 0.001) in discordant MZ twins. Additionally,* PPARG* expression was positively correlated with body mass (*r* = 0.714, *P* = 0.047) and height (*r* = 0.762, *P* = 0.028).

The concordant MZ twins demonstrated negative correlation of the VO_2_max with body mass (*r* = −0.290, *P* = 0.027), BMI (*r* = −0.472, *P* = 0.001), waist circumference (*r* = −0.426, *P* = 0.001), and sum of skinfolds (*r* = −0.739, *P* = 0.001). However, in MZ group, there was no correlation between* PPARG* mRNA expression with anthropometric characteristics and VO_2_max (Table 3).

**Figure 1:**
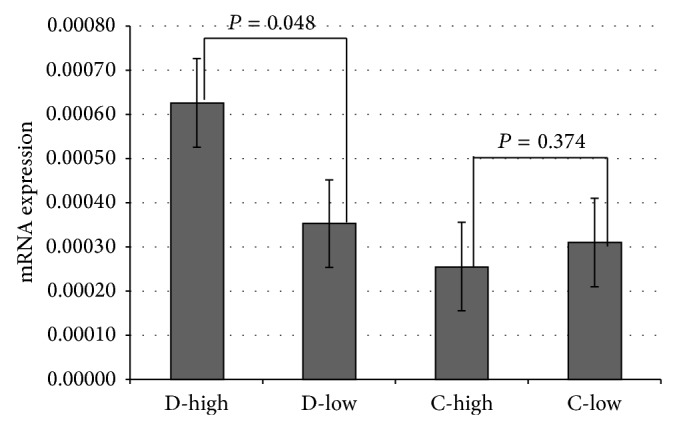
*PPARG* mRNA expression in blood total leukocytes from VO_2_max discordant and concordant MZ twins. mRNA expression is shown as ΔCt values in relation to* GAPD* (reference gene). Results between low and high VO_2_max within discordant (D-low versus D-high) and concordant (C-low with C-high) pairs were compared by paired Wilcoxon test.

Regarding the* PPARG* mRNA expression, it was observed that discordant cotwins with the high VO_2_max values (D-High) had 1.78-fold greater* PPARG* mRNA expression when compared to cotwins with the low VO_2_max values (D-low) (*P* = 0.048) (Figure 1). In the concordant group, cotwins with low VO_2_max values (C-low) had 1.22-fold greater PPARG mRNA expression when compared to cotwins with high VO_2_max (C-high). However, there was no significant difference (*P* = 0.374) (Figure 1).

## 4. Discussion

The major finding from this study indicates that VO_2_max is capable of affecting* PPARG* mRNA expression in VO_2_max-discordant MZ twins regardless of genetic background. The results showed 30.6% higher VO_2_max values in cotwins discordant for VO_2_max (D-High) compared with cotwins with low VO_2_max values (D-Low), but this difference is not associated with differences in anthropometric variables between twin's pars discordant and concordant.

The correlation analysis was performed for anthropometric characteristics with VO_2_max and* PPARG* mRNA expression in MZ twin's pairs with discordant and concordant for VO_2_max. According to results for VO_2_max-discordant MZ twins, the VO_2_max values were negatively correlated with sum of skinfolds and strong positive correlation with* PPARG* mRNA expression. The* PPARG* mRNA expression was positively correlated with body mass and height. For VO_2_max-concordant MZ twins, the VO_2_max values were negatively correlated with body mass, BMI, waist circumference, and sum of skinfolds. Interestingly for this group, there was no correlation between* PPARG* mRNA expression with anthropometric characteristics and VO_2_max.

In previous studies, there was correlation between obesity (BMI) and VO_2_max (*r* = −0.88, *P* < 0.05) in young subjects [26] and a strong positive correlation between the ratio of* PPARG (splice variants y2/y1)* mRNA and the BMI (*r* = 0.70, *P* < 0.001) in obese patients [27]. The expression of* PPARG* mRNA* (splice variants y2)* is increased in adipose tissue in obese men and women, and their direct correlation with BMI is unknown but it may be related to the expansion of adipose tissue mass [27].

The* PPARG* mRNA expression in circulating monocytes from healthy individuals was increased after participating in a program of cycling exercise that caused a significant increase in VO_2_max [18]. Significant increase in leukocyte mRNA expression for* PPARG* was observed in healthy but previously sedentary individuals that participated in an 8-week low-intensity exercise program consisting of walking 10,000 steps, three times a week [28]. We have demonstrated that children and adolescents with high VO_2_max showed higher* PPARG* mRNA expression. This result was reinforced by the strong correlation between VO_2_max and* PPARG* mRNA expression. Although the cardiorespiratory fitness of the MZ twin pairs was evaluated only once (cross-sectional study), the method used to measure VO_2_max (indirect calorimety based in gas exchange) is more accurrate than the physical activity evaluation using questionnaires and surveys, which have been questioned [29]. The absolute value of VO_2_max is one of the indices of an individual's cardiorespiratory fitness to transport oxygen to working muscles. Additionally the evidence suggests that physically active children have significantly better cardiorespiratory fitness levels than inactive children [30, 31].

In an animal study, the effect of acute and endurance exercise training on mRNA expression pattern of the different PPARs and* ppara* coactivator-1 alpha (PGC-1*α*) in muscles that largely rely on either glycolytic (plantaris) or oxidative (soleus) metabolism was analyzed [17]. Moreover,* pparγ* mRNA levels were the lowest in skeletal muscle which is the most responsive to changes in physical activity levels.

In spite of the important contribution of this study, there are some limitations such as the cut-off values of the discordant cardiorespiratory fitness and timing of blood sampling for RNA extraction. The cardiorespiratory fitness (based on studies of familial aggregation and twins) shows significant genetic effects (50–67%) [32, 33]; our results should be interpreted with caution. Among the assumptions of the study with twins, one of them admits that the phenotype carries great genetic background when showing low variance between MZ twins. In classical studies with MZ twin pairs, discordance is defined using criteria based on literature in which phenotype is a condition usually acquired [34]. However, even in studies in which variance allows establishing discordance between identical twins (i.e., one twin is diabetic/obese/hypertensive/sedentary and the other is not), environmental and/or genetic factors that may have significantly influenced this discrepancy are still questioned [35, 36]. In addition, it was observed that the behavior of the cardiorespiratory fitness is unstable during life, being influenced by several factors such as age, gender, and physical activity level. It should be emphasized that, besides the improvement promoted by physical exercise on VO_2_max, the period corresponding to childhood and adolescence is marked by growth and gradual development of the cardiorespiratory system components that determine VO_2_max (lungs, heart, and muscles) [37]. In this sense, possible discrepancies in VO_2_max in this age group in genetically identical individuals would be more likely to be due to environmental factors (physical activity and/or exercise). In other words, the determinants that distinguish aerobic fitness of a MZ twin pair compared to his cotwin could be due to environmental factors because, in this model, the changes observed in VO_2_max as a function of growth and development of the various systems would be under genetic control. Considering the increase in VO_2_max caused by growth, reducing aerobic capacity at this stage is less likely than increasing it. Thus, ignoring errors from evaluation, evaluator, and measurement equipment, it is possible that discordant cotwin pair with higher VO_2_max in this study is actually more active than his correspondent cotwin.

We should also highlight that differences in the* PPARG* mRNA expression observed between discordant MZ twins (D-high versus D-low) may be influenced by the timing of blood sample collection. However it is noteworthy that concordant twins (C-high versus C-low) underwent the same protocol, but did not show variability in* PPARG* expression. There is evidence showing that the* PPARG* mRNA expression increases significantly (1.5- to 2.5-fold) up to 3 hours after exercise, but returning to basal levels within 24 h of exercise [18]. Reinforcing this information, it was observed that the mRNA of the PPAR family of transcription factors is sensitive to exercise training in skeletal muscle but is not particularly sensitive to acute bouts of endurance exercise. These findings would suggest that changes in the PPAR mRNA expression are most likely mediated by the accumulation of repeated bouts of exercise as opposed to single bouts of activity [17].

Regarding the cut-off value of 10 mL·kg^−1^·min^−1^ adopted to establish discordance between MZ twin pairs in this study. In a longitudinal study with MZ and DZ twin pairs discordant for cardiorespiratory fitness (VO_2_ peak of 26.4 ± 4.9 versus 32.5 ± 5.5 mL·kg^−1^·min^−1^), the researchers used lower cut-off values (≈6 mL·kg^−1^·min^−1^) and emphasized that the differences within twin pairs were sufficient to identify metabolic disorders [38]. In another study, researchers adopted 9% as cut-off value to determine discordances (mean of 18 ± 10%) in the VO_2_max values of MZ twins. The results revealed that the more and less active twins showed, respectively, VO_2_max values of 50.9 ± 5.1 versus 43.4 ± 6.7 mL·kg^−1^·min^−1^ [39]. It is noteworthy that the relative difference between twins with higher and lower VO_2_max (discordant) in this study ranged from 16.9% to 42.1%, with mean of 30.6 ± 9.5% (data not shown). It is also noteworthy that the absolute average of these differences was 13.5 ± 3.7 mL·kg^−1^·min^−1^ for discordant pairs and approximately 7.5 mL·kg^−1^·min^−1^ for the study of Hannukainen et al. [39].

In conclusion, the results of this study suggest that the cardiorespiratory fitness evaluated by VO_2_max parameter may modulate the* PPARG* mRNA expression in childhood and adolescence, independently of the genetic background. Considering that the* PPARG* expression regulates lipid and glucose metabolism, insulin action and adipocyte differentiation the effect of cardiorespiratory fitness on* PPARG* may contribute to the prevention of future metabolic diseases in adults such as type 2 diabetes.

## Figures and Tables

**Table 1:** Primers and probes used for mRNA expression by qPCR.

Gene	Primers	Amplicon size
*PPARG*	5′ GCAAAGGCGAGGGCG 3′	82 bp
	5′ CCCATCATTAAGGAATTCATGTCAT 3′	
	5′ FAM CAGACAAATCACCATTCG MGB 3′	

*GAPD *	5′ GGAAGGTGAAGGTCGGAGTCA 3′	229 bp
	5′ CTGGAAGATGGTGATGGGATTTC 3′	
	5′ VIC TCAGCCTTGAGGGTGC MGB 3′	

Note: bp: base par.

**Table 2:** Anthropometric characteristics of discordant and concordant MZ twins pairs for VO_2_max.

Variables	Discordant pairs (*n* = 9)^†^		Concordant pairs (*n* = 29)^†^		*F*	*P*
	D-high	D-low	C-high	C-low		
Age (years)	13.9 ± 2.2		14.2 ± 2.1		—	—
*n* (♂/♀)	18 (8/10)		58 (20/38)		—	—
VO_2_max	45.9 ± 10.0	32.4 ± 10.6^a^	42.4 ± 9.2^b^	38.8 ± 9.8	3.690	0.016
Body mass (kg)	46.4 ± 9.0	46.2 ± 8.7	52.1 ± 14.1	53.7 ± 13.6	1.291	0.284
Height (cm)	155.7 ± 11.5	156.4 ± 11.0	158.8 ± 10.6	159.1 ± 10.6	0.335	0.800
BMI (kg/m^2^)	18.9 ± 1.4	18.7 ± 1.5	20.4 ± 4.2	21.0 ± 4.4	1.290	0.284
Waist (cm)	66.5 ± 4.4	65.7 ± 5.0	70.6 ± 11.7	71.8 ± 10.9	1.218	0.310
TR + SB (mm)	18.9 ± 6.2	19.4 ± 6.1	28.2 ± 16.6	29.7 ± 17.0	1.924	0.124

VO_2_max: maximal oxygen uptake (mL·kg^−1^·min^−1^); BMI: body mass index; TR + SB: sum of skinfold triceps and subscapular. D-high and D-low: discordant pairs; C-high and C-low: concordant pairs. ^†^VO_2_max intrapair difference ≥10 mL·kg^−1^·min^−1^; data are shown as mean ± SD and compared by ANOVA test, with Bonferroni test for multiple comparisons. ^a^ *P* < 0.025, D-low versus D-high; ^b^ *P* < 0.050, D-low versus C-high.

**Table 3:** Correlation of VO_2_max and *PPARG* mRNA expression with anthropometric characteristics in MZ twins pairs.

Variables	Discordant pairs (*n* = 9)		Concordant pairs (*n* = 29)	
	VO_2_max	*PPARG *	VO_2_max	*PPARG *
Body mass (kg)	0.064	0.714^**^	−0.290^*^	−0.163
Height (cm)	0.086	0.762^*^	0.126	0.135
BMI (kg/m^2^)	−0.109	0.214	−0.472^**^	−0.172
Waist (cm)	0.046	0.323	−0.426^**^	−0.201
TR + SB (mm)	−0.540^*^	−0.357	−0.739^**^	−0.243
VO_2_max/*PPARG *	0.952^**^		0.309	

VO_2_max: maximal oxygen uptake (mL·kg^−1^·min^−1^); BMI: body mass index; TR + SB: sum of skinfold triceps and subscapular. Spearman's rank correlation tests ^**^ *P* = 0.01 level (2-tailed); ^*^ *P* = 0.05 level (2-tailed).
